# Deciphering the Molecular Signatures Associated With Resistance to *Botrytis cinerea* in Strawberry Flower by Comparative and Dynamic Transcriptome Analysis

**DOI:** 10.3389/fpls.2022.888939

**Published:** 2022-05-27

**Authors:** Guilin Xiao, Qinghua Zhang, Xiangguo Zeng, Xiyang Chen, Sijia Liu, Yongchao Han

**Affiliations:** Hubei Key Laboratory of Vegetable Germplasm Enhancement and Genetic Improvement, Institute of Industrial Crops, Hubei Academy of Agricultural Sciences, Wuhan, China

**Keywords:** strawberry (*fragaria* × *ananassa*), flower, *Botrytis cinerea*, RNA sequencing, resistance-related genes

## Abstract

Gray mold caused by *Botrytis cinerea*, which is considered to be the second most destructive necrotrophic fungus, leads to major economic losses in strawberry (*Fragaria* × *ananassa*) production. *B. cinerea* preferentially infects strawberry flowers and fruits, leading to flower blight and fruit rot. Compared with those of the fruit, the mechanisms of flower defense against *B. cinerea* remain largely unexplored. Therefore, in this study, we aimed to unveil the resistance mechanisms of strawberry flower through dynamic and comparative transcriptome analysis with resistant and susceptible strawberry cultivars. Our experimental data suggest that resistance to *B. cinerea* in the strawberry flower is probably regulated at the transcriptome level during the early stages of infection and strawberry flower has highly complex and dynamic regulatory networks controlling a multi-layered defense response to *B. cinerea*. First of all, the higher expression of disease-resistance genes but lower expression of cell wall degrading enzymes and peroxidases leads to higher resistance to *B. cinerea* in the resistant cultivar. Interestingly, CPKs, RBOHDs, CNGCs, and CMLs comprised a calcium signaling pathway especially play a crucial role in enhancing resistance by increasing their expression. Besides, six types of phytohormones forming a complex regulatory network mediated flower resistance, especially JA and auxin. Finally, the genes involved in the phenylpropanoid and amino acids biosynthesis pathways were gene sets specially expressed or different expression genes, both of them contribute to the flower resistance to *B. cinerea*. These data provide the foundation for a better understanding of strawberry gray mold, along with detailed genetic information and resistant materials to enable genetic improvement of strawberry plant resistance to gray mold.

## Introduction

*Botrytis cinerea* is a necrotrophic fungal pathogen that causes gray mold disease in more than 1,400 plant species, including almost all vegetable and fruit crops (Elad et al., 2016). This polyphagous pathogen is regarded as the second most destructive phytopathogen globally, as it is found worldwide and damages fruit, flower, and leaf both before and after harvest (Dean et al., 2012). Data suggest that gray mold disease causes $10 billion to $100 billion in losses per year (Weiberg et al., 2013).

Plants have evolved a multi-layered defense system against *B. cinerea*. First, the plant cell wall acts as a physical barrier to *B. cinerea* infection (Underwood, 2012; Blanco-Ulate et al., 2014). Next, once the pathogen penetrates cell walls, plants detect this attack and trigger signaling pathways that induce defense responses at the plant cell walls (Hematy et al., 2009). Plant pattern recognition receptors (PRRs) such as receptor-like kinases (RLKs) and receptor-like proteins (RLPs) recognize pathogen-associated molecular patterns (PAMPs) and host damage-associated molecular patterns (DAMPs), activating PAMP-triggered immunity, key transcriptional regulators in the defense against *B. cinerea* (Mengiste, 2012; Lai and Mengiste, 2013). The signals from these RLKs and RLPs are then transduced to mitogen activated protein kinase (MAPK)-dependent and/or -independent cascades and activate *WRKY33*, resulting in the up-regulation of genes involved in camalexin biosynthesis (Birkenbihl et al., 2012; Zhou et al., 2020). Plant hormones such as salicylic acid (SA), jasmonic acid (JA), ethylene (ET), and abscisic acid (ABA) also contribute to the plant resistance to *B. cinerea* (AbuQamar et al., 2017). However, to date, little information has been obtained about the effects of *B*. *cinerea* infection on these processes in strawberry (*Fragaria* × *ananassa*) (Underwood, 2012; González et al., 2013; Li et al., 2013; Petrasch et al., 2019; Lu et al., 2020), a popular small fruit crop with short production cycles, extremely high nutrition, and good flavor.

*Botrytis cinerea* preferentially infects strawberry flowers and fruits, leading to flower blight and fruit rot, which are the two most important causes of yield and economic losses. Studies have demonstrated that *B. cinerea* inoculum primarily enters the strawberry flower organs; the infected petals, stamens, and calyxes and then facilitate primary infection in fruits (Petrasch et al., 2019). The previous studies have focused primarily on the interaction of on *B. cinerea* with strawberry fruit (Liang et al., 2018; Xiong et al., 2018; Haile et al., 2019) while paying little attention to its interaction with the flower.

Moreover, the researches on resistance mechanisms have been hampered by the lack of fully resistant strawberry resources (Bestfleisch et al., 2015). As documented by Bristow et al. (1986), the levels of resistance to *B*. *cinerea* varies between strawberry cultivars as detailed as follows: Fungal growth in stamens appears to be strongly inhibited—so that the fungus never reaches the receptacle—in some cultivars, but not in others.

In our previous work, we established a method to evaluate *B. cinerea* resistance in strawberry flower and found different levels of resistance in different cultivars (data not shown). Nonetheless, the molecular signatures have not been deciphered until now. The recent research has demonstrated that next-generation RNA sequencing (RNA-seq) can serve as a powerful tool to elucidate the transcriptional reprogramming induced by biotic and abiotic stresses (Kou et al., 2018; Guo et al., 2019). The RNA-seq has been used to study the interactions between *B*. *cinerea* and plant hosts including the model plants Arabidopsis (Windram et al., 2012) and tomato (Blanco-Ulate et al., 2013; Smith et al., 2014) and the non-model plants lettuce (De Cremer et al., 2013), cucumber (Kong et al., 2015), grape (Haile et al., 2017), and kiwi (Zambounis et al., 2020). However, this approach has so far provided little information about *B. cinerea* infection of strawberry (Xiong et al., 2018; Haile et al., 2019), and particularly strawberry flower.

In our previous study, we identified two cultivars with different levels of resistance to *B. cinerea*: the resistant Yanli (Y) and the susceptible H. In the present study, we conducted a time–series and comparative transcriptome analysis of these two strawberry cultivars designed to uncover the molecular mechanisms of strawberry resistance to *B*. *cinerea*. By analyzing the transcriptomes of these cultivars at eight time points, we obtained a global view of gene expression changes involved in *B*. *cinerea* resistance in the strawberry flower. Our findings enhance the understanding of molecular interactions between *B*. *cinerea* and strawberry flower and provide gene resources for improving strawberry resistance.

## Results

### Flowers of Two Strawberry Cultivars Showed Different Degrees of *B. cinerea* Resistance

**Figure 1:**
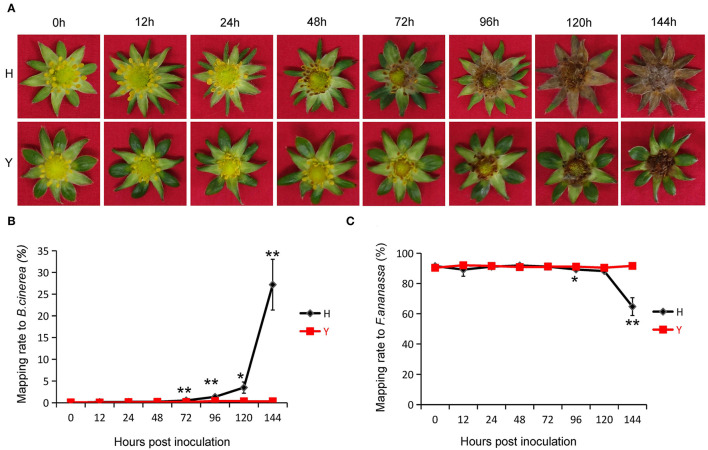
Resistance of strawberry flower to *Botrytis cinerea*. **(A)** Disease symptoms of two cultivars, Y and Hongyan (H), displaying resistance and susceptibility to *B. cinerea*. **(B)** The ratio of successfully mapped reads to the genome of *B. cinerea* at 12, 24, 48, 72, 96, 120, and 144 h post-inoculation (hpi). An average mapping rate (%) of a single time point has been presented. Error bars indicate standard error within three biological replicates. Statistically significant differences between cultivars for each time point are indicated by one asterisk (*p* < 0.05) or two asterisks (*p* < 0.01). **(C)** The ratio of successfully mapped reads to the genome of cultivated strawberry at all eight time points.

Although strawberry petals could be colonized by conidia of *B. cinerea*, the quick abscission of petals after blossoming reduced the probability of infection by this means. Therefore, we first removed petals from the *in vitro* flowers of two strawberry cultivars, Y and H, and then inoculated these apetalous flowers with *B. cinerea* to examine their resistance. The two cultivars responded differently: cultivar H was susceptible to *B. cinerea*, showing mild symptoms at 24 h post inoculation (hpi) and typical gray mold symptoms at 48 hpi, whereas cultivar Y was resistant, with no detectable symptoms at 48 hpi, mild symptoms at 72 h, and typical gray mold symptoms only after 96 hpi (Figure 1A). The fact that the obvious differences in symptoms were observed from 48 hpi indicated that resistance to *B. cinerea* in the strawberry flower is probably regulated at the transcriptome level during the early stages of infection.

To investigate the transcriptome dynamics of strawberry flowers after *B. cinerea* inoculation, we performed RNA-seq with total RNA isolated from the flowers of the two cultivars at eight widely spaced time points, 0 (before inoculation), 12, 24, 48, 72, 96, 120, and 144 hpi with three independent biological replicates at each time point. A total of 397.55 GB clean data were obtained from all 48 samples. The clean reads were mapped to the *B. cinerea* and *Fragaria* × *ananassa* genome (Camarosa, FAN_r1.1) with HISAT2 (Kim et al., 2015) and the mapping rates were calculated, respectively. As expected, the mapping rate of clean reads aligned to *B. cinerea* genome increased and the mapping rate to the strawberry genome decreased with the extension of inoculation time (Figures 1B,C). There was no significant difference between Y and H until 72 hpi. At 144 hpi, the mapping ratio to the *B. cinerea* genome had increased to 27.19 ± 5.84% in H and 0.34 ± 0.99% in Y and the mapping ratio to the strawberry genome had decreased to 64.73 ± 5.90% in H and 91.63 ± 0.71% in Y.

### Global Transcriptomic Changes of Strawberry Flower After Infection by *B. cinerea*

We assembled the mapped reads and quantified them in fragments per kilobase of transcript length per million mapped reads (FPKM) using StringTie (Pertea et al., 2015). A total of 90,767 genes were identified including 87,797 known genes and 2,970 previously identified genes. Among them, ~86% genes expressed (FPKM > 0.1) in at least one of the 16 samples.

**Figure 2:**
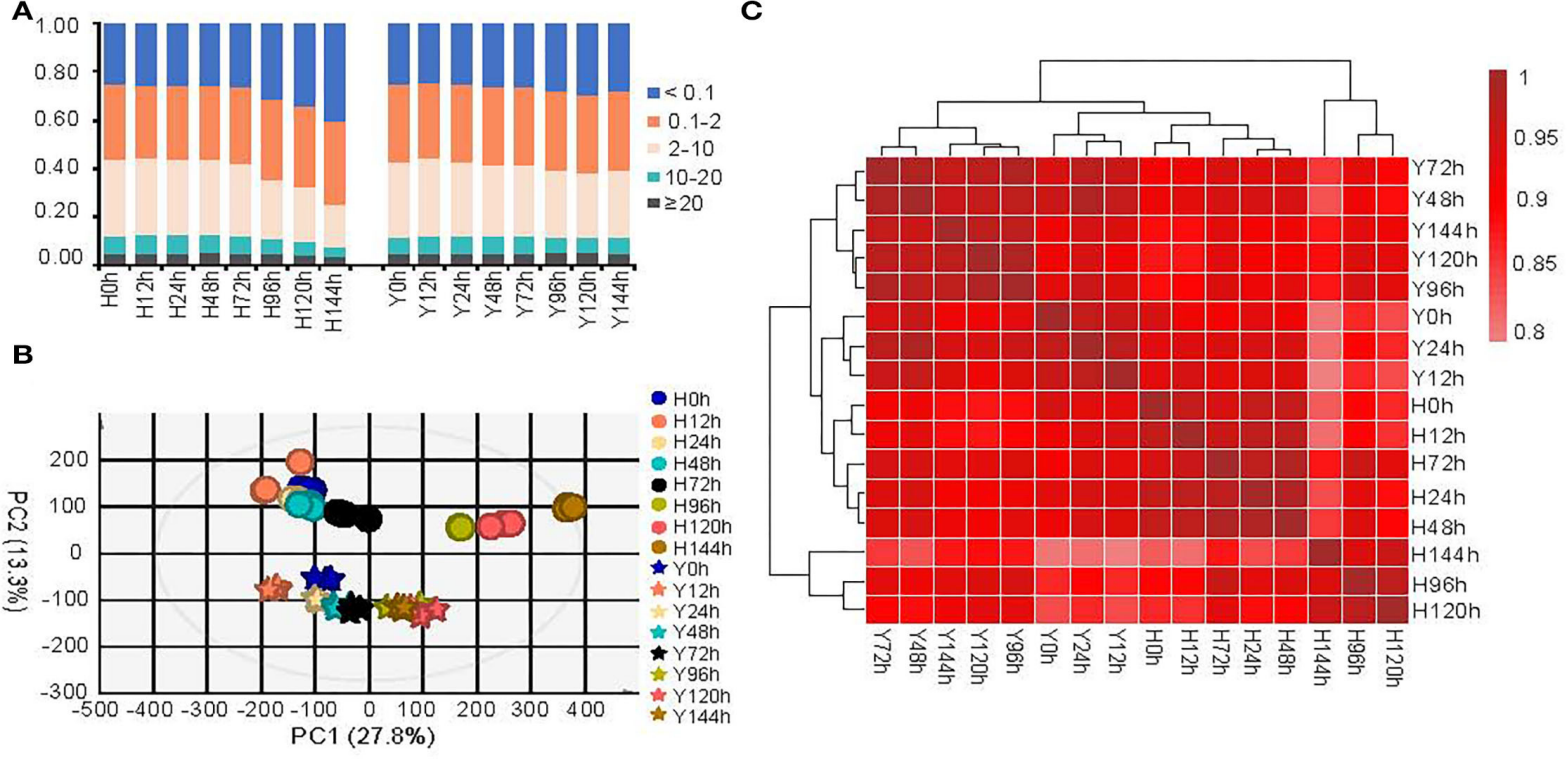
Global views of gene expressions for the resistant strawberry cultivar Y and susceptible cultivar H at all eight time points. **(A)** Percentage of genes expressed at different expression levels in the two cultivars (based on FPKM). The PCA **(B)** and the hierarchical clustering **(C)** based on Spearman's correlation coefficient of average FPKM values. The circle and asterisk represent the cultivar H and Y in PCA, respectively.

The percentage of genes expressed ranged from 75.0% (at 0 hpi) to 59.5% (at 144 hpi) in H and 75.3% (at 12 hpi) to 70.5% (at 120 hpi) in Y (Figure 2A). About 3.8–5.1% of genes showed very high (FPKM ≥ 20) expression both before and after infection by *B. cinerea* in both cultivars. The number of genes exhibiting high (10 ≤ FPKM > 20), moderate (2 ≤ FPKM > 10), and low (0.1 ≤ FPKM > 2) expression were similar in all samples, except the susceptible cultivar H showed lower overall expression after 72 hpi, which was in accordance with the mapping rates of clean reads (Figure 2A). Overall, the total proportion of genes showed very high, high, and moderate expression was greatest at 12 hpi, followed by 24 hpi, revealing transcriptome-induced resistance to *B. cinerea* at the early infection stages.

To inspect the global difference between H and Y before and after *B. cinerea* infection, we performed principal component analysis (PCA) and hierarchical clustering based on Spearman's correlation coefficient of the average FPKM values for all genes (Figures 2B,C). We observed substantial differences between the two cultivars, with the H and Y samples being divided into two groups in the PCA (Figure 2B). However, there was also a highly significant correlation between H and Y, as evidenced by correlation coefficients >0.78 (Figure 2C). Interestingly, the results of PCA as well as hierarchical clustering showed that the transcriptomes of H at 96, 120, and 144 hpi differed substantially from those at the other time points, in accordance with the mapping rate for *B. cinerea*. Furthermore, cultivar H showed severe symptoms after 96 hpi. Consequently, we conducted a further differential gene expression analysis at 0–72 hpi. Replicate 3 of H12 was removed because it was separated from the other two replicates in PCA and had relatively low correlation with the other samples in H (Supplementary Table 1).

Twelve genes were selected for validation of their expression by qRT–PCR (Supplementary Figure 1). The results of Pearson correlation analysis demonstrate a significant correlation (*r* = 0.468, *p* < 0.05) between qRT–PCR and RNA-seq. The transcript levels of *EDS1, CML41, PYL4, PAL1, ARF5, GH3.5, BGLU12, 4CL1, SGT1*, and *PP2CA* were high in the resistant cultivar Y, whereas the transcript levels of *WRKY33* and *RPM1* were high in the susceptible cultivar H.

### Resistant Cultivar Y Exhibited More Sophisticated Expression Patterns

**Figure 3:**
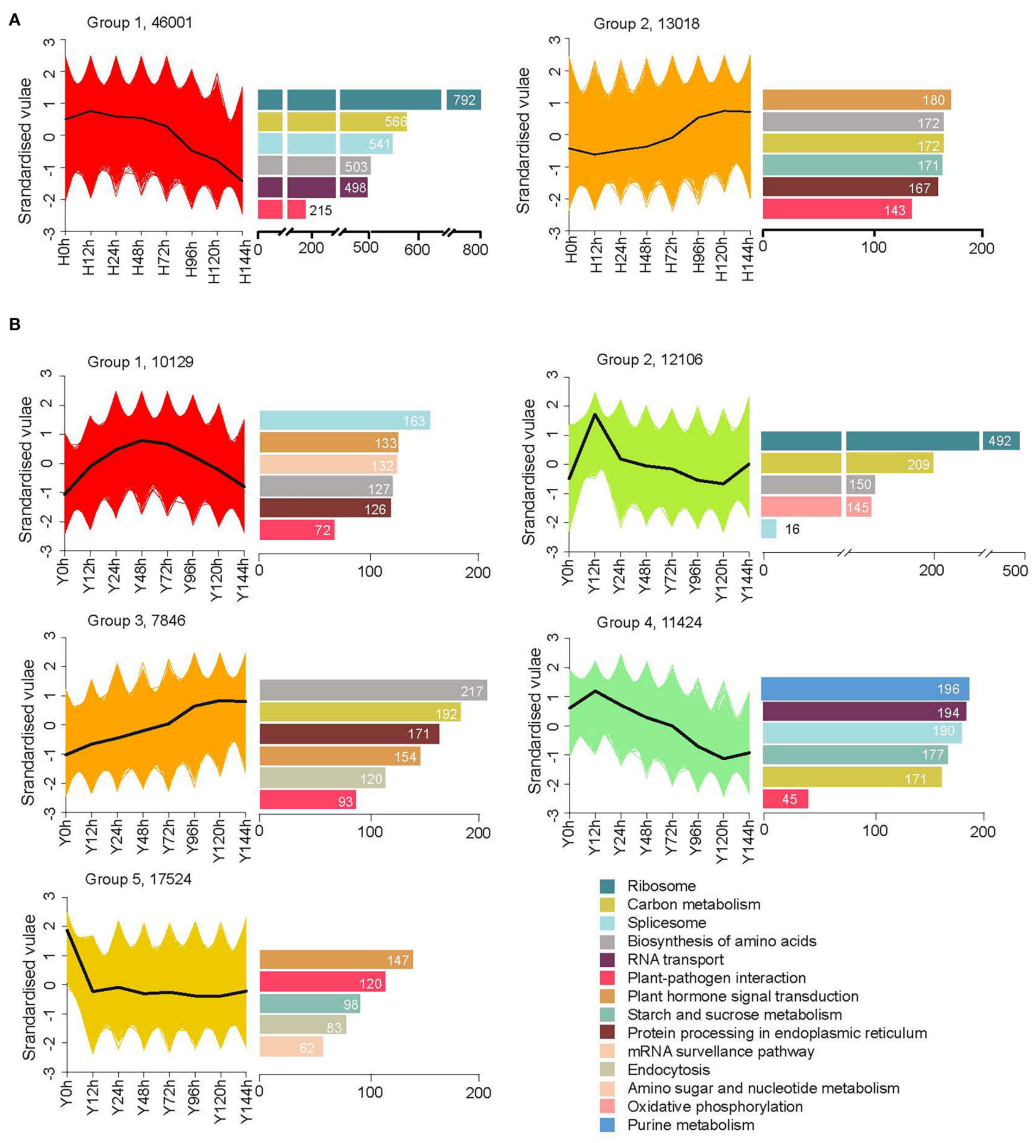
More sophisticated expression patterns in the resistant cultivar Y **(B)** than the susceptible cultivar H **(A)**. Cluster assignments of genes with FPKM ≥ 1 are shown in the left graph and, correspondingly, the top five KEGG enriched pathways as well as the “plant–pathogen interaction” pathway are shown in the right bar charts.

To identify expression clusters for each cultivar, we applied *K*-means clustering with the time series gene expression datasets. The cluster assignments of genes with FPKM ≥ 1 and the top five Kyoto Encyclopedia of Gene and Genomes (KEGG)-enriched pathways as well as the “plant–pathogen interaction” pathway are displayed in Figure 3. A total of 59,122 genes were clustered into five groups in the resistant cultivar Y, but only two groups in susceptible cultivar H, indicating that Y has a more complex response than H at the transcriptome level. As the time since inoculation increased, the expression levels in H decreased for 77.9% (46,001) and increased for 22.1% (13,018) of the genes. In Y, one-third of the genes (17,524) belonged to group 5, which was characterized by up-regulation at 12 hpi followed by down-regulation until 120 hpi, and the other genes were almost equally divided between groups 1 and 4.

Approximately 60% of the genes in the “plant–pathogen interaction” pathway were clustered in the down-regulated group 1, and the other ~40% were clustered in the up-regulated group 2 in H. Again, Y exhibited a more range of diverse expression changes than H. Over one-third of “plant–pathogen interaction” genes were clustered in group 3, where genes were drastically down-regulated at 12 hpi and then showed slight fluctuations in expression, with a maximum at 24 hpi. One-quarter and one-fifth of genes were in groups 2 and 1, respectively, and smaller numbers of genes were assigned to groups 5 and 4. The diverse expression clusters of Y are likely to account for resistance to *B. cinerea* for *in vitro* strawberry flower.

### Five KEGG Pathways Enriched of Gene Sets Specially Expressed (SEGs) as Well as Different Expression Genes (DEGs)

**Figure 4:**
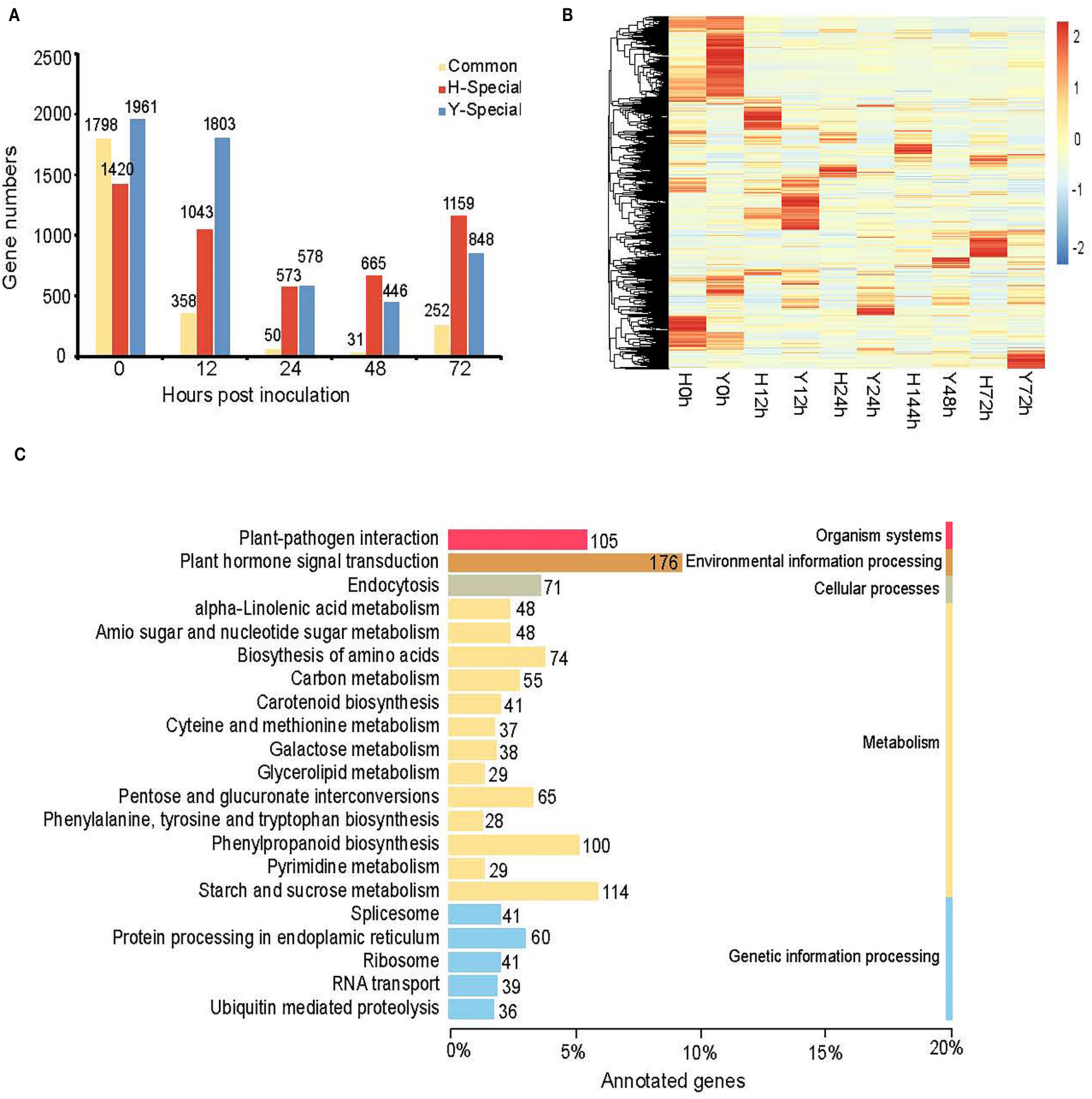
Specially expressed genes at 0, 12, 24, 48, and 72 hpi for Y and H. **(A)** Bar graph showing the number of specifically expressed genes specifically and commonly in Y and H at each time point. **(B)** Heatmap showing the expression profile of specifically expressed genes at different time points in both cultivars. The color scale represents *Z*-score. **(C)** The top 21 enriched KEGG pathways of specifically expressed genes for five time points in Y and H.

We identified gene sets specially accumulated at 0–72 hpi by applying a stage-specificity (SS) scoring algorithm to uncover the transcriptional differences that characterized each time point for resistant cultivar Y and susceptible cultivar H and potentially mitigated *B. cinerea* infection. Based on the criterion SS ≥ 0.5, we identified 11,950 SEGs for two cultivars at five time points. Among them, 8,125 and 7,349 were included for Y and H, respectively (Figure 4). The trend of changes in SEG numbers in Y was coincident with that in H as described in the following: First, both decreased and then increased. However, the turning point occurred later in the resistant cultivar Y (48 hpi) than in the susceptible cultivar H (24 hpi). In Y, 628 (7.7%) and 477 (5.9%) genes showed high transcript accumulation at 24 and 48 hpi, respectively. In H, 8.5% (623) genes were specifically expressed at 24 hpi and 9.5% (696) at 48 hpi.

A large difference between Y and H was observed at 12 hpi, and the number of SEGs for Y was more than that of H from 0 hpi to 24 hpi, whereas the number of SEGs for H was more than Y at 48 and 72 hpi, indicating that the induced resistance for strawberry flower was acquired at the early infection stages (Figure 4A). A visual display of the expression profiles for all these genes in both cultivars is shown in Figure 4B. Furthermore, we analyzed the KEGG (http://www.genome.jp/kegg/) pathway of SEGs at five time points and identified the enrichment pathways (Figure 4C). The pathway “plant hormone and signal transduction” was the most enriched, followed by “starch and sucrose metabolism,” “plant–pathogen interaction,” “phenylpropanoid biosynthesis,” and “biosynthesis of amino acids.” The numbers of SEGs in these five pathways were 176, 114, 105, 100, and 74, respectively.

**Figure 5:**
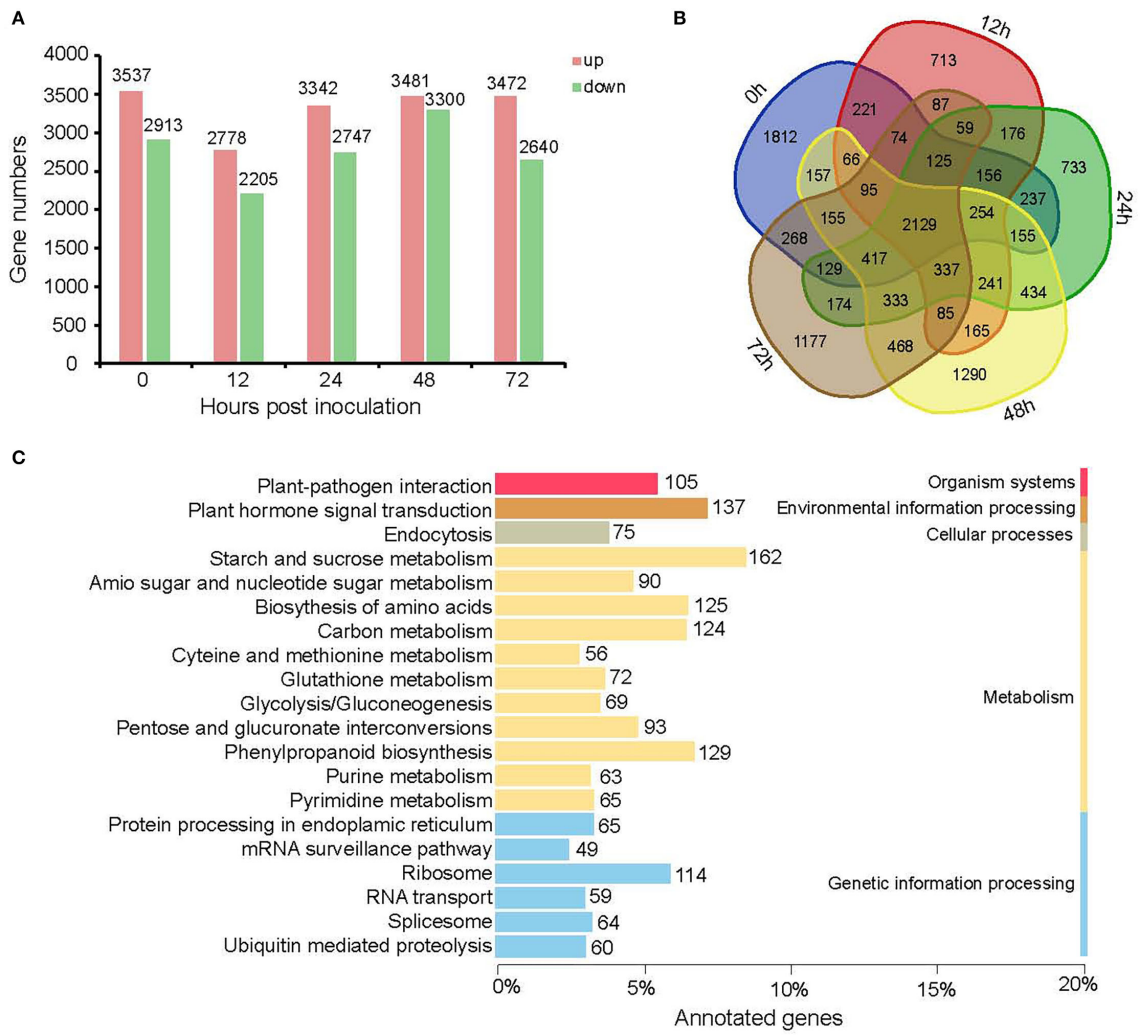
Differential gene expressions in Y as compared with H at different time points. **(A)** The column chart shows the number of up-regulated (light coral bars) and down-regulated (spring green bars) genes at each time point. **(B)** Venn diagrams showing the common and specific DEGs for five time points. **(C)** The top 20 enriched KEGG pathways of DEGs between Y and H for five time points.

We further identified DEGs between the resistant cultivar Y and the susceptible cultivar H for each time point from 0 to 72 hpi (Figure 5). A total of 12,922 genes were differentially expressed between Y and H at one or more of the five time points.

The number of genes up-regulated in Y was greater than the number down-regulated in Y at all five time points, indicating that the resistance is likely acquired through the activation of a larger number of defense-related genes (Figure 4A). For four of the five time points (0, 24, 48, and 72 hpi), the number of DEGs varied from 6,089 to 6,781, whereas it was only 4,983 at 12 hpi. A Venn diagram of common and specific DEGs shows 337 common DEGs for the four inoculation time points but not for the “no inoculation” time point (0 hpi) (Figure 4B). The top seven enriched KEGG pathways of all DEGs at the five time points were “starch and sucrose metabolism,” “plant hormone and signal transduction,” “phenylpropanoid biosynthesis,” “biosynthesis of amino acids,” “carbon metabolism,” “ribosome”, and “plant–pathogen interaction.” The number of DEGs in each pathway was 162, 137, 129, 125, 124, 114, and 105, respectively (Figure 4C). Among them, “plant hormone and signal transduction,” “starch and sucrose metabolism,” “plant–pathogen interaction,” “phenylpropanoid biosynthesis,” and “biosynthesis of amino acids” were enriched for both SEGs and DEGs, indicating that these pathways may be involved in resistance to *B. cinerea* for strawberry flower. There were 798 genes included in these five pathways (Supplementary Table 2).

### Transcriptional Regulatory Network Controlling Strawberry Flower Resistance to *B. cinerea*

**Figure 6:**
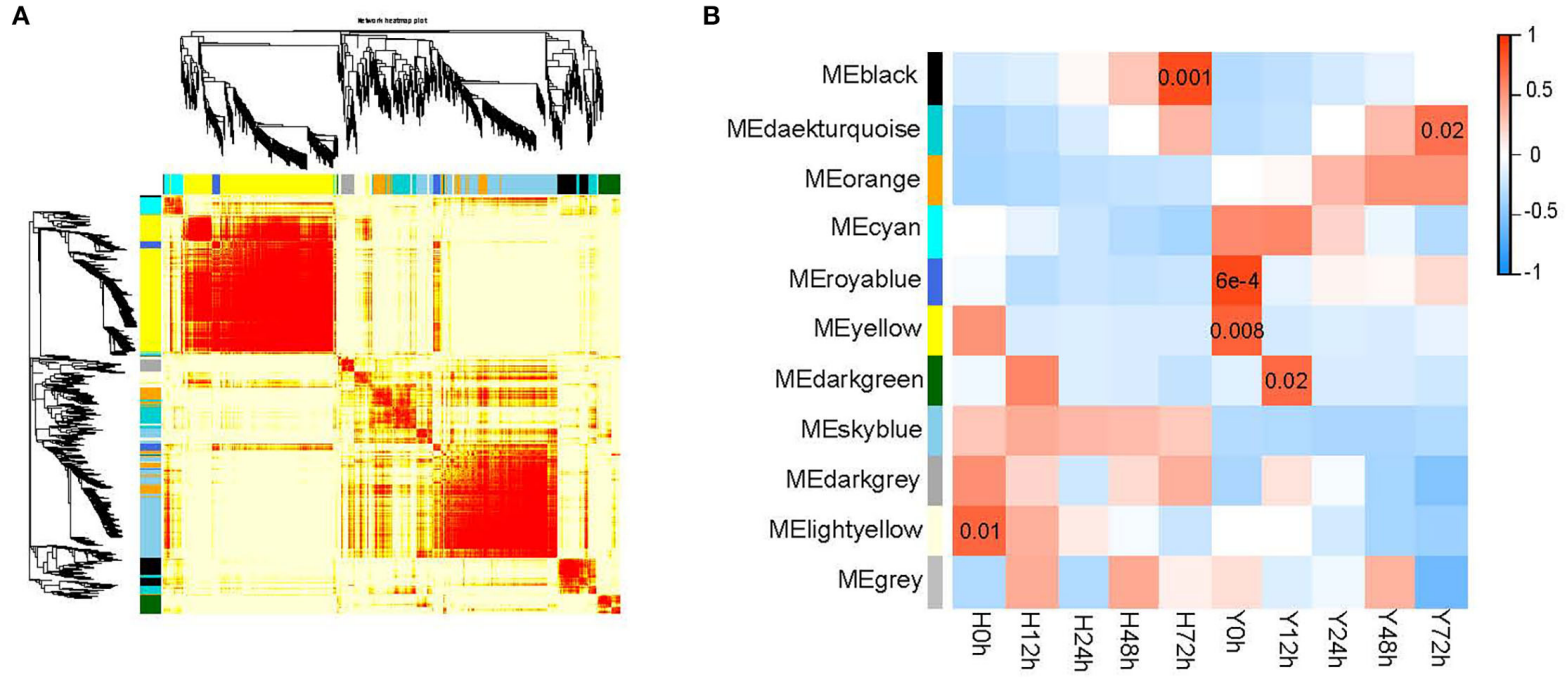
The construction of gene co-expression modules in both cultivars for five time points. **(A)** Hierarchical clustering tree (dendrogram) of genes based on co-expression network analysis in Y and H. The branches correspond to modules of highly interconnected genes. Each module was assigned by a unique color; with ungrouped genes colored gray. **(B)** Correlation between gene co-expression modules (row) and resistance to *B. cinerea* for two cultivars at five time points (column). The correlation coefficients and the *p*-value are presented at the top and bottom of the corresponding boxes. The color legend for modules is indicated on the left.

**Figure 7:**
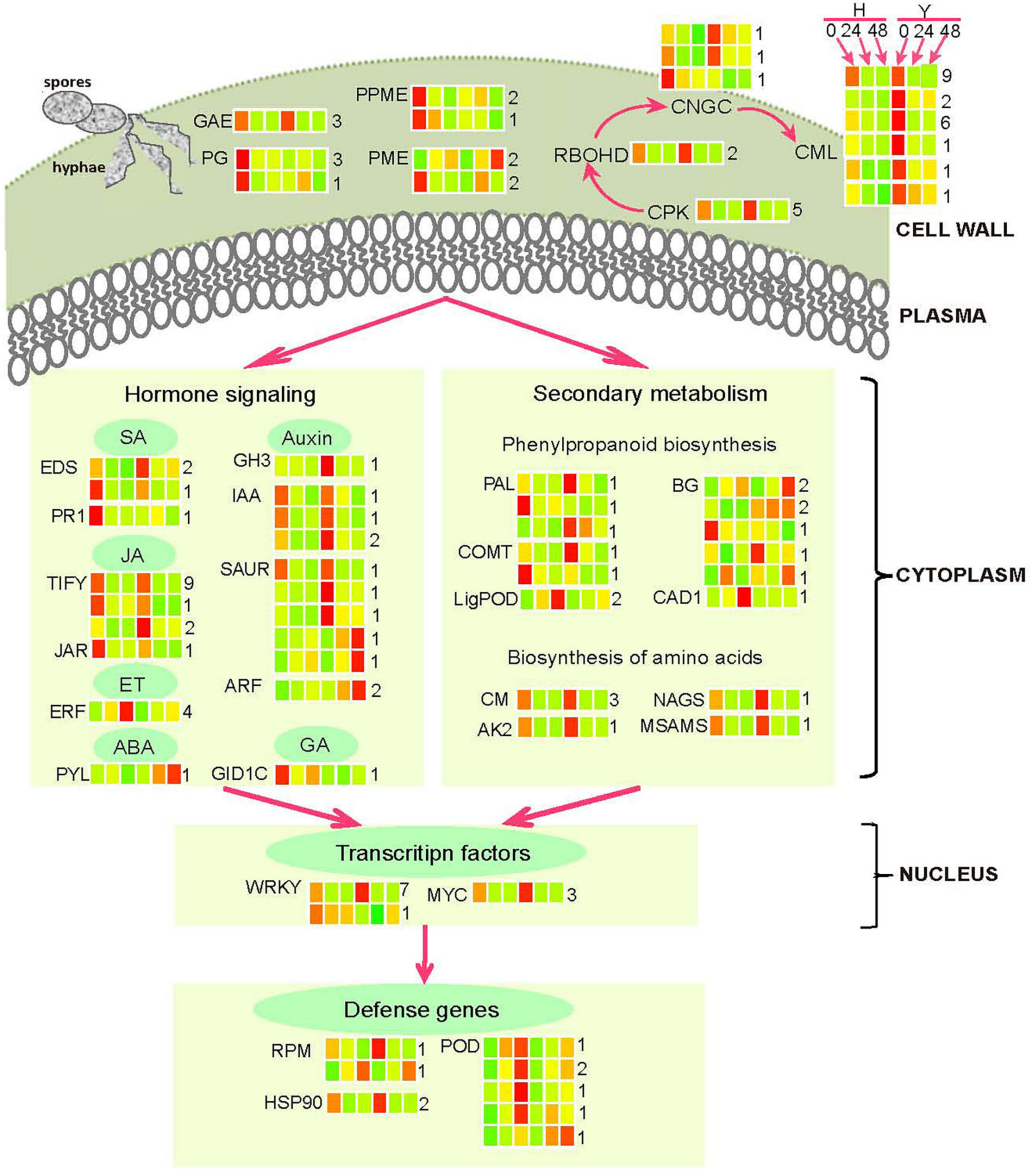
General model summarizing *B. cinerea* resistance mechanisms in strawberry flowers. The red coloring represents up-regulation, green coloring represents down-regulation, and yellow indicates no significant change in heatmaps (infected leaves at 0, 24, and 48 hpi of the resistant cultivar Y and the susceptible cultivar H). The full name and gene accession numbers of differentially regulated genes are listed in Supplementary Table 2. The numbers behind the heat maps represent the numbers of genes in the indicated family.

To elucidate the gene co-expression network that arises in response to the infection of *B. cinerea* in strawberry flower, a weighted gene co-expression network analysis (WGCNA) was utilized within DEGs and SEGs (Figure 6). A total of 22,496 genes were included in the WGCNA. Finally, 3,748 genes were grouped into 10 modules that were defined using color codes, along with a gray module representing the remaining uncorrelated genes (Figure 6A). The most genes (1,202) were contained in the yellow module, followed by the skyblue (910) and orange (356) modules. A few genes were contained in the darkgray (98) and royalblue (131) modules. Among the 10 modules, 6 were significantly (*p* < 0.05) correlated with *B*. *cinerea* resistance (Figure 6B). The royalblue module was the most significant one, followed by the black and yellow modules. The other three closely related modules were the lightyellow, darkgreen, and darkturquoise modules. Consequently, 152 genes in the associated modules in WGCNA overlapped with genes in the “plant–pathogen interaction,” “plant hormone and signal transduction” starch and sucrose metabolism,” “phenylpropanoid biosynthesis,” and “biosynthesis of amino acids” pathways (Supplementary Table 2). A clear regulation of these pathways and candidate genes were displayed in Figure 7.

In the plant resistance to the necrotrophic pathogen *B. cinerea*, the transcription factor *WRKY33* and *MYC2* were well-studied genes. Here, we identified eight WRKY transcription factors including *WRKY33* and three *MYC2* genes. There were another three well-known disease-resistance proteins including the R protein for resistance to *Pseudomonas syringae* pv. *maculicola* 1 (*RPM1*) and the heat shock protein 90 (*HSP90*) conferring biotic stress tolerance. All these disease-resistance genes improve *B. cinerea* resistance in strawberry flower by increasing the expression: highest in the resistant cultivar Y before inoculation except *WRKY34* and one gene annotated as *RPM1*. Additionally, 14 cell wall-related genes including 7 pectinesterases (PMEs/PPMEs), 4 polygalacturonases (PGs), and 3 UDP-glucuronate 4-epimerases (GAEs) were highly expressed in the susceptible cultivar H before inoculation which may lead to the sensitivity. Different from these genes mentioned here, five out of the six peroxidases (PODs) were induced by *B. cinerea*. The expression of them increased more rapidly in the susceptible cultivar H than the resistant cultivar Y leading to the higher expression in H than Y at 24 and 48 hpi. Our results suggest calcium signaling plays crucial role in strawberry flower resistance to *B*. *cinerea*. A total of 30 genes including 5 calcium-dependent protein kinases (CPKs), 2 respiratory burst oxidase homolog proteins D (RBOHDs), 3 cyclic nucleotide-gated ion channels (CNGCs), and 20 calcium-binding proteins (CMLs) had the same expression pattern as the disease-resistance genes except one *CNGC*.

Furthermore, our results also reveal that plant defense to *B. cinerea* is mediated by hormonal regulatory networks. First, an SA marker gene pathogenesis-related genes 1 (*PR1*) was specially accumulated in the susceptible cultivar H before inoculation. Also, *EDS1* was another gene in the SA signaling, one of them showed the same expression module as *PR1* whereas two of them highly expressed in the resistant cultivar Y before inoculation. Except for this gene mentioned here, *MYC2*, there were 12 TIFY family genes as well as one JA–amido synthetase (*JAR1*) belonging to the JA biosynthesis pathway genes. Three of them were differentially expressed between two cultivars. Besides, a total of 12 genes in the auxin pathway belonged to the 3 gene families as follows: Auxin–responsive proteins (SAURs and IAAs), auxin response factors (ARFs), and indole-3-acetic acid-amido synthetases (GH3s). More than half of the auxin-signaling genes displayed the same expression module as disease-resistance genes. Furthermore, the expression of four ethylene-responsive transcription factors (ERFs) increased with time after inoculation and higher in H than Y. The last but not the least, two receptors, each in the ABA and GA signaling pathway, namely, ABA receptor (PYL) and gibberellin receptor (GID1C) had different expression levels between cultivars. The *PYL* had the highest expression in the resistant cultivar in Y at 48 hpi while the *GID1C* had the highest expression in H before inoculation.

Finally, genes in the phenylpropanoid biosynthesis and amino acids biosynthesis pathways were involved in the resistance to *B. cinerea* in strawberry flower. There were six amino acid-biosynthesis genes, including three chorismate mutases (CMs), one aspartokinase (*AK2*), one amino acid acetyltransferase (*NAGS2*) and one S-adenosylmethionine synthase 2 (*MSAMS2*). All these amino acid–biosynthesis genes showed the expression trend consistent with disease-resistance genes. Genes in the phenylpropanoid biosynthesis pathway mainly comprised of lignin biosynthesis genes, such as β-glucosidases (BGs), phenylalanine ammonia-lyases (PALs), caffeic acid 3-O-methyltransferases (COMTs), lignin-forming anionic peroxidases (LigPODs), and cinnamyl alcohol dehydrogenase (*CAD*). Four and three of them reached the highest expression before inoculation in the resistant cultivar Y and the susceptible H, respectively. The other six genes increased their expression after inoculation and showed the highest expression at 48 hpi.

## Discussion

### In Response to Infection, the Resistant Strawberry Cultivar First Up-Regulated Disease-Resistance Genes and Down-Regulated Cell Wall-Degrading Enzymes and Peroxidases

Three genes that are known be involved in the interaction between *B. cinerea* and host plants, *EDS1* (Bhandari et al., 2019; Baggs et al., 2020; Lapin et al., 2020), *WRKY33* (Birkenbihl et al., 2012; Zhou et al., 2020) and *MYC2* (Lorenzo et al., 2004) were identified by WGCNA analysis with the dynamic and comparative transcriptome data. These genes were highly expressed in the resistant cultivar Y before inoculation in addition to *RPM1* and *HSP90*, disease-resistance proteins for other pathogens (Huang et al., 2014; El Kasmi et al., 2017; Ul Haq et al., 2019). Therefore, it seems likely that most disease-resistance genes in strawberry flower were prepared to resist invading pathogens before inoculation. In addition, we found several genes involved in hormone and Ca^2+^ signaling showed similar expression patterns. We speculate that this phenomenon may exist specially at the flower stage accompanied by the transition from vegetative to reproductive development. However, more experiments are needed to confirm this possibility.

The plant cell wall serves as the first line of defense against pathogen penetration. Our results indicate that the susceptible cultivar H increases the expression of cell wall-degrading enzymes including PGs, PMEs/PPMEs, and GAEs, leading to the flower sensitivity. The previous studies have documented that these enzymes mediate immunity by degrading cell walls or affecting cell wall integrity and structure (Bethke et al., 2014, 2016; Lionetti et al., 2017). The pectinesterase AtPME17 and three pectinesterase inhibitors have been proven to be involved in Arabidopsis resistance to *B. cinerea* (Lionetti et al., 2017; Del Corpo et al., 2020). Same as the cell wall-degrading enzymes, PODs showed higher expression in the susceptible cultivar H than Y, indicating that PODs negatively regulate *B. cinerea* resistance. This is consistent with a previous study that illustrated that PODs play a crucial role in the generation of H_2_O_2_ or other ROS in the immune response (Daudi et al., 2012).

In conclusion, disease-resistance genes were up-regulated in the resistant cultivar Y but cell wall-degrading enzymes and peroxidases were down-regulated, which is supported by previous studies. Unfortunately, few PRRs important for PTI were found though serine/threonine-protein kinases were included in Supplementary Table 2.

### The Calcium Signaling Pathway Plays a Crucial Role in the Resistance to *B. cinerea* in Strawberry Flower

Although Ca^2+^ is well known to serve as a second messenger in cell signaling, how Ca^2+^ encodes complex information with high specificity in various signaling processes, such as development and biotic interactions, remains an area of intense study. The most striking result to emerge from our study was that 30 genes belonging to the calcium signaling pathway accounted for about 20% of all the 152 genes selected by WGCNA. Although little attention has been focused on the calcium signaling pathway genes in the interaction between strawberry and *B*. *cinerea*, our knowledge on the Ca^2+^ dependent-immunity mechanisms advanced recently (Liang et al., 2018; Xiong et al., 2018; Haile et al., 2019). Considering the model plant Arabidopsis as an example, active BOTRYTIS-INDUCED KINASE1 (BIK1) and CPKs phosphorylate Ca^2+^-bound RBOHD to boost ROS production. The CaM-gated CNGC2–CNGC4 complex is then activated, leading to the sustained cytosolic Ca^2+^ elevation and the relay of Ca^2+^-dependent immunity (Tian et al., 2020). Interestingly, 30 calcium signaling pathway genes found in this study belonged to the CPKs, RBOHDs, CNGCs, and CMLs. Moreover, these genes displayed the same expression module as disease-resistance genes: highly expressed in the resistant cultivar Y. The CMLs had the largest number (20) of genes. The expression of *CML41* in the resistant cultivar Y was 51.2 and 27.9, as shown by the FPKM value in H at 0 hpi and was 2.7 times higher than that in H at 24 hpi, which was validated by qRT–PCR. Consistent with our results, recent research has shown that two CALMODULIN-LIKE genes (*CML46* and *CML47*) and *cbp60a* (CALMODULIN-BINDING PROTEIN60) contribute to two different modes of negative regulation of immunity (Lu et al., 2018). Same as CMLs, RBOHDs, and CPKs are Ca^2+^ sensors decoding the spatial and temporal calcium concentration changes in the cytoplasm to convey Ca^2+^ signals into specific cellular responses (Lu et al., 2018; Bredow and Monaghan, 2019; Lee et al., 2020). The CNGCs mediate calcium entry and provide a critical link between the PAMP–PRR complex and calcium-dependent immunity programs in the PTI signaling pathway (Tian et al., 2019). According to these data, we infer that CPKs, RBOHDs, CNGCs, and CMLs comprise a calcium signaling pathway that plays a crucial role in enhancing resistance to *B*. *cinerea* by their increased expression.

### Resistance to *B. cinerea* Is Mediated by Hormonal Regulatory Networks

The most enriched pathway we identified in this study is the “plant hormone and signal transduction” pathway, which involves six types of phytohormones, namely, SA, JA, auxin, ET, ABA, and gibberellic acid (GA). These results corroborate with previous findings (AbuQamar et al., 2017), suggesting an essential role of phytohormones in the plant response to *B*. *cinerea*. Also, SA is well known to regulate plant defense response to pathogens including *B*. *cinerea*. This research identified the following two well-known genes in the SA signaling pathway: *EDS1* and *PR1* (Breen et al., 2017). A marker gene in the SA pathway, *PR1*, was highly expressed in the susceptible cultivar H, indicating negative regulation of SA in strawberry resistance. This finding was confirmed by earlier work (Ferrari et al., 2003; Breen et al., 2017). The genes in the JA and auxin pathways were the most and second most enriched with 12 and 13 genes, respectively, implying primary roles in the resistance. Twelve TIFY genes in the JA pathway that have been proven to be involved in the resistance to bacterial blight in rice (Yamada et al., 2012) and powdery mildew in *Vitis vinifera* (Yu et al., 2019) displayed a similar expression module as *MYC2*, a transcription factor in this pathway. The majority of auxin-related genes were differentially expressed between two cultivars. These results provide further support for our hypothesis that auxin contributes to defense response against necrotrophic pathogens (Nafisi et al., 2015). IAA and GH3 have been reported to regulate plant disease-resistance in cassava (Fan et al., 2020) and Arabidopsis (Zhang et al., 2007), respectively. By contrast, four ERFs were induced by inoculation in this study. The previous research demonstrated that ERF1 confers resistance to several necrotrophic fungi in Arabidopsis (Berrocal-Lobo et al., 2002), and that ERF099 is an important regulator involved in *B*. *cinerea* resistance in rose petal (Li et al., 2020). Finally, *PYL4* and *GID1C*, the ABA and GA receptor genes, have been demonstrated to mediate Arabidopsis immune responses toward necrotrophic and biotrophic pathogens (Garcia-Andrade et al., 2020) and rice resistance to blast fungus (Tanaka et al., 2006), respectively. These results suggest that six types of phytohormones form a complex regulatory network that mediates flower resistance to *B*. *cinerea*, especially JA and auxin.

### The Phenylpropanoid and Amino Acid Biosynthesis Pathways Are Involved in the Resistance to *B*. *cinerea*

We also found that genes in the phenylpropanoid biosynthesis pathway were enriched. Similarly, Dong and Lin (2021) documented that phenylpropanoid metabolism contributes to plant development and the interplay between plants and the environment, including biotic and abiotic stresses. We found 15 genes, including 7 BGs, 3 PALs, 2 COMTs, 2 LigPODs, and a *CAD1*, belonging to the phenylpropanoid biosynthesis pathway. The BGs serve as detonators of plant chemical defense against pathogens and herbivores (Morant et al., 2008). The FaBG3, which encodes β-glucosidase in *F. ananassa*, has been shown to regulate fruit ripening, dehydration stress, and *B. cinerea* fungal infection in strawberries *via* modulation of ABA homeostasis and transcriptional regulation of ripening-related genes (Li et al., 2013). The previous reports have indicated that lignin is deposited at the infection site to inhibit the penetration and growth of pathogens (Mutuku et al., 2019; Lee et al., 2020; Xiao et al., 2021), and relevant genes, such as PALs, *CAD*, and COMTs, have been confirmed, through functional genomics experiments, to be associated with disease-resistance (Tonnessen et al., 2015; Li et al., 2019; Hoch et al., 2021).

The amino acid metabolic pathways have been considered to be integral to the plant immune system (Zeier, 2013). We found that the CMs were DEGs and *AK2* was a SEG. Overexpression of CM lead to increased resistance to *Xanthomonas oryzae* in rice (Jan et al., 2020) and *B. graminis* f. sp. *Hordei* in barley (Hu et al., 2009). Additionally, the loss-of-inhibition allele of *AK2* exhibited strong resistance to *Hyaloperonospora arabidopsidis* (*Hpa*) (Stuttmann et al., 2011). Combining our findings with the previous reports, we conclude that the phenylpropanoid and amino acid biosynthesis pathways are involved in the resistance to *B. cinerea*.

## Materials and Methods

### Plant Materials and Pathogen Inoculation

Two strawberry cultivars, Y and H, with contrasting resistance to *B. cinerea* were acquired. Strawberry plants were grown in the field during the winter season (September–April) in two consecutive years. The field management was conducted according to local practices to ensure normal crop growth. *In vitro* inoculation of *B. cinerea* was performed when plants reached peak flowering.

*Botrytis cinerea* strain BC-1 was isolated in March 2017 and maintained at 4°C in darkness. At 2 or 3 weeks before inoculation, BC-1 was subcultured on potato dextrose agar (PDA) in the dark at 20 ± 1°C under an alternating 1-h darkness/12-h light photoperiod to produce conidia. Spore inoculums were prepared by harvesting conidia in water and filtered through double gauze to remove hyphae. Then, the concentration of the pathogen suspension was adjusted to 10^6^ spores ml^−1^ through counting on a hemacytometer.

The stamens of flowers with petals removed were spray inoculated so that the suspensions were evenly spaced over the stamens. To investigate the transcriptome dynamics, time–series inoculations were employed. The whole flowers without petals were harvested before inoculation (0 hpi) as well after inoculation by *B. cinerea* at 12, 24, 48, 72, 96, 120, and 144 hpi, immediately frozen in liquid nitrogen, and then later subjected to RNA extraction and sequencing. There were three biological replicates for each time point for both cultivars.

### The RNA Sequencing, Read Mapping, and Differential Gene Expression Analysis

Total RNA was extracted from each sample (~100 mg) using RNAprep Pure Plant Kit (Tiangen Biotech, Beijing, China) following the manufacturer's instructions. The library products were prepared for sequence analysis *via* NEBNext UltraTM RNA Library Prep Kit for Illumina (NEB, USA). An Illumina NovaSeq 6000 instrument was used for sequencing, and paired-end reads were generated. These raw reads were processed by removing reads containing the adapter sequences, reads with more than 5% poly-N, and low-quality reads (reads with more than 50% of low-quality bases of quality value ≤ 5) to obtain clean reads. At the same time, Q20, Q30, GC content, and sequence duplication level of the clean data were calculated. A total of 397.55 GB high-quality clean reads (average 5.95 GB reads from each sample) were generated for from 48 samples and all the downstream analyses were based on these clean data.

Filtered reads were mapped to the octoploid strawberry genome *Fragaria* × *ananassa* available at https://datadryad.org/resource/10.5061/dryad.b2c58pc (Edger et al., 2019) and *B. cinerea B05.10* from NCBI by HISAT2 (Kim et al., 2015) and then assembled by StringTie (Pertea et al., 2015). The gene expression level was calculated as FPKM by StringTie. The genes exhibiting a difference in expression level of at least 2-folds, with corrected *p*-value after adjusting with false discovery rate ≤ 0.01, were considered to be DEGs by DESeq2. The genes specifically expressed (SEGs) at any time point in both cultivars were identified *via* the SS scoring algorithm of Zhan et al. (2015), which compares the expression of a gene at a given time point with its maximum expression level at other time points. The genes with SS score ≥ 0.5 were regarded as specifically expressed at one time point. All raw reads were deposited to the National Center for Biotechnology Information Sequence Reads Archive (SRA) with accession number PRJNA761556. Supplementary Table 4 presented the sample name and their corresponding accession number.

### Gene Annotation and Functional Enrichment Analysis

All assembled genes were annotated based on the following database: Nr (http://ftp.ncbi.nih.gov/blast/db/, NCBI non-redundant protein sequences), Nt (NCBI non-redundant nucleotide sequences), Pfam (http://pfam.xfam.org/, Protein family), KOG/COG (http://www.ncbi.nlm.nih.gov/KOG/, http://www.ncbi.nlm.nih.gov/COG/, Clusters of Orthologous Groups of proteins), Swiss–Prot (http://www.uniprot.org/, A manually annotated and reviewed protein sequence database), KO (http://www.genome.jp/kegg/, KEGG Ortholog database), and GO (http://www.geneontology.org/, Gene Ontology). On the basis of annotation, we conducted gene ontology (GO) enrichment and KEGG pathway enrichment analysis for both DEGs and SEGs. The GO enrichment analysis of the differentially expressed genes (DEGs) was implemented by the GOseq R packages based on Wallenius non-central hyper-geometric distribution (Young et al., 2010), which can adjust for gene length bias in DEGs. The KEGG pathway enrichment analysis of DEGs was implemented with KOBAS software (Mao et al., 2005).

### Co-expression Regulatory Network Construction by WGCNA

The coexpression regulatory network was constructed by WGCNA (Langfelder and Horvath, 2008) for both DEGs and SEGs based on their correlation patterns. A total of 22,496 genes were used for analysis. The soft threshold was set to 30 to make the network fit to a scale-free topology. The GO and KEGG pathway enrichment analyses were performed for each module as introduced here. In addition, we detected the association of each module with the phenotype of resistance to *B. cinerea* (0 and 1 were designated as resistant and susceptible phenotypes, respectively, for Y and H). A positive correlation indicated that genes in a module have higher expression in the resistant cultivar than the susceptible one.

### Absolute Quantitative qRT–PCR Analysis

Absolute qRT–PCR experiments were applied for validating the results of RNA-seq. The total RNA for each sample were extracted as described here. The gene-specific primers designed using Primer Express (v3.0) software are listed in Supplementary Table 3. There were three biological replicates for each time point and cultivar, with three technical replicates for each biological replicate. The transcript level of each gene was normalized by comparison with the internal control gene, housekeeping gene gene11892 (Gao et al., 2020), and fold change was calculated according to the 2^−^ΔΔCT method.

## Data Availability Statement

The datasets presented in this study can be found in online repositories. The names of the repository/repositories and accession number(s) can be found below: National Center for Biotechnology Information (NCBI) BioProject database under accession number PRJNA761556.

## Author Contributions

YH conceived and supervised the project. GX performed the inoculation experiment, qRT-PCR, analyzed the transcriptomic data, and wrote the draft manuscript. QZ initialed the inoculation experiment and revised the manuscript. XZ, XC, and SL managed plant propagation and field operation for plant growth as well as the inoculation experiment. All authors have read and approved the submitted version.

## Funding

This work was supported by Grants from the National Natural Science Foundation of China (31701882), National Key Research and Development Program of China (2018YFD1000300), Hubei Province Key R&D Program (2021BBA099), and Agricultural Science and Technology Innovation Center Program of Hubei Province (2019-620-000-001-08).

## Conflict of Interest

The authors declare that the research was conducted in the absence of any commercial or financial relationships that could be construed as a potential conflict of interest.

## Publisher's Note

All claims expressed in this article are solely those of the authors and do not necessarily represent those of their affiliated organizations, or those of the publisher, the editors and the reviewers. Any product that may be evaluated in this article, or claim that may be made by its manufacturer, is not guaranteed or endorsed by the publisher.
