# Supplementary figures and images for: Deciphering the Molecular Signatures Associated With Resistance to Botrytis cinerea in Strawberry Flower by Comparative and Dynamic Transcriptome Analysis
Source: Front Plant Sci. 2022 May 27;13:888939. doi: 10.3389/fpls.2022.888939 (PMC9198642; doi:10.3389/fpls.2022.888939)

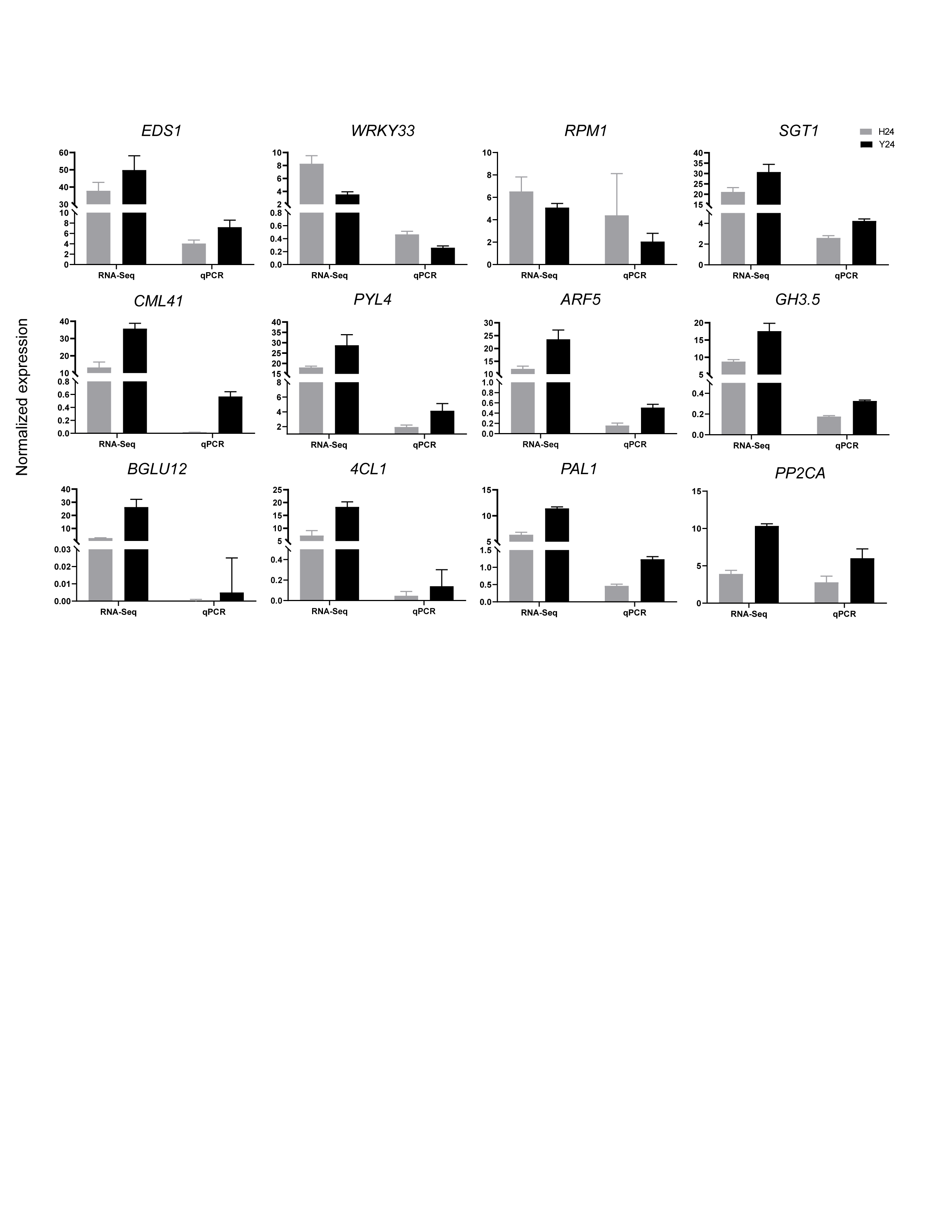

Supplement: Supplementary file 5 [file Image_1.TIF]
